# Can an Universal School-Based Social Emotional Learning Program Reduce Adolescents’ Social Withdrawal and Social Anxiety?

**DOI:** 10.1007/s10964-023-01840-4

**Published:** 2023-08-17

**Authors:** Vanda Sousa, Patrícia Ribeiro Silva, Ana Maria Romão, Vítor Alexandre Coelho

**Affiliations:** 1 Centro de Investigação em Psicologia para o Desenvolvimento, Universidade Lusíada Porto, Rua de Moçambique, 21 e 71, 4100-348 Porto, Portugal; 2 Académico de Torres Vedras, Travessa do Quebra-Costas, 9, 2560-703 Torres Vedras, Portugal

**Keywords:** Social withdrawal, Social anxiety, School climate, Social and emotional learning

## Abstract

There is a lack of studies analyzing if universal school-based Social and Emotional Learning programs can reduce social withdrawal and social anxiety. This study analyzed the effectiveness of one such program on those variables, and the role of individual school climate perceptions. In this nationwide study, 704 seventh to eighth-grade Portuguese students (*M*_*age*_ = 12.96, *SD* = 1.09, 48% girls), of which 215 (30.6%) in the comparison group, were assessed at pretest, post-test, and follow-up seven months later. Analyses showed positive intervention results in self- and teacher-reported social withdrawal and social anxiety. Regarding school climate, intervention group students with more positive teacher-student relationships benefitted more from program participation in social anxiety. These results support the program’s effectiveness for addressing social withdrawal and social anxiety.

## Introduction

Mental health difficulties during adolescence are a cause for concern due to their lifelong effects on well-being, general health, and the ability to establish and maintain healthy relationships (Clarke et al., 2021; Greenberg et al., 2017). Currently, one of most promising and effective interventions for the promotion of mental health in adolescents is social and emotional learning (Clarke et al., 2021), with extensive evidence that social and emotional learning programs are effective in enhancing social emotional competencies (Durlak et al., 2022, Taylor et al., 2017). However, there is scarce evidence of a direct effect on mental health difficulties, such as social withdrawal and social anxiety, which became an even more prominent issue in the context of the recent COVID-19 pandemic, that had strong negative effects upon students’ mental health and, specifically, on students’ social withdrawal and social anxiety (Hawes et al., 2021). Furthermore, there is an increasing consensus that schools represent a natural setting for the implementation of universal interventions for mental health promotion (Clarke et al., 2021; Greenberg et al., 2017), because they are not subject to the common barriers that hinder interventions offered outside of schools, such as high levels of dropout and low levels of attendance (Coelho & Sousa, 2018; Van de Sande et al., 2019). The use of schools as the setting for these interventions highlights the relevance of accounting for school climate as facilitator or deterrent of SEL program’ effectiveness (Domitrovich et al., 2022; Stalker et al., 2018). This study aims to analyze the effectiveness of a SEL program, the Positive Attitude Upper Middle School (PAUMS) program, on social anxiety and social withdrawal, using a nationwide sample to expand the generalization of the results over previous studies with the PAUMS program. Furthermore, this study is the first to analyze how individual perceptions of school climate may hinder or facilitate SEL program’ effectiveness on social withdrawal and social anxiety.

### Social Anxiety and Social Withdrawal

Throughout adolescence, not only social anxiety but also social withdrawal is associated with difficult peer relationships, with socially anxious and withdrawn students displaying greater difficulties in cooperation and other pro-social behaviors (Miers et al., 2013). Both social anxiety and social withdrawal represent major problems in adolescence that interfere with personal and social functioning and cause deep distress and unease (Hebert et al., 2013; Smith et al., 2017) and, therefore, the prevention of social anxiety and social withdrawal in adolescence has attracted growing interest from researchers and practitioners (Clarke et al., 2021; Stallard et al., 2014).

Social anxiety is defined as persistent anxiety concerning social situations in which potential scrutiny from others may occur (Berger et al., 2017). A social anxiety continuum exists during adolescence, with concern or apprehension about a particular social situation or situations at the lower end of the continuum and intense social anxiety at the upper end, which could lead to the avoidance of a wide range of social situations (Blöte et al., 2015). Therefore, social anxiety is a psychosocial problem that represents an important factor for understanding interpersonal behavior (Pabian & Vandebosch, 2016; Van den Eijnden et al., 2014).

Previous studies have reported various negatives consequences of high levels of social anxiety in children and adolescents: lower self-esteem (Goméz-Ortiz et al., 2018), lower peer acceptance (Scanlon et al., 2020), higher peer rejection (Blöte et al., 2012), greater difficulties in the development of same-sex friendships and romantic relationships (Chiu et al., 2021; Hebert et al., 2013), and higher levels of peer victimization and cybervictimization (Chiu et al., 2021; Coelho et al., 2022). Furthermore, socially anxious youth have an increased risk of academic underachievement (Scanlon et al., 2020), because social anxiety interferes with their ability to form and maintain positive peer support networks. Specifically, this negative effect on their formation of peer support networks leads to a decrease in their social engagement in academia, which, then, affects their academic performance.

Social withdrawal refers to an adolescent’s isolation from known and unknown peers through the constant display of solitary behavior over time and across situations (Rubin et al., 2009). Social withdrawal is often presented as being negative overall and related to loneliness, since socially withdrawn adolescents spend more time alone than their peers (Bamps et al., 2022; Smith et al., 2017). As these individuals are on the periphery of the social scene, they often miss out on the positive developmental opportunities that result from peer interaction (Bowker & White, 2021). Furthermore, the isolation behavior displayed by socially withdrawn adolescents could cause their peers to perceive them negatively; consequently, social withdrawal places adolescents at risk of developing emotional problems like anxiety and depression (Gazelle & Faldowski, 2019; Smith et al., 2017) and has been associated with peer rejection (Bowker & White, 2021).

### Social and Emotional Learning (SEL)

Social and Emotional Learning (SEL) has been defined by the Collaborative for Academic, Social, and Emotional Learning (CASEL, 2022) as “the process through which all young people and adults acquire and apply the knowledge, skills, and attitudes to develop healthy identities, manage emotions and achieve personal and collective goals, feel and show empathy for others, establish and maintain supportive relationships, and make responsible and caring decisions.”. Social and Emotional Learning promotes the development of social and emotional competencies, which are associated with social, behavioral, and academic outcomes which predict important life outcomes in adulthood (Domitrovich et al., 2017).

Currently, there is strong evidence that SEL programs—mainly universal preventive interventions conducted in schools— have a positive impact on social and emotional competence, academic performance, self-image, and pro-social behavior and attitudes toward the self, school, and others (Coelho & Sousa, 2018; Durlak et al. 2022; Taylor et al., 2017). Furthermore, in the long term, greater social and emotional competence can lead to better mental health (Clarke et al., 2021; Zhang et al., 2023) and well-being (Greenberg et al., 2017). Specifically, there is evidence that universal prevention programs (specifically SEL programs) may reduce anxiety levels (Stallard et al., 2014; Teubert & Pinquart, 2011), although one study found that the relationship between social and emotional competencies and internalizing problems was mediated by resiliency (Colomeischi et al., 2022). Furthermore, those interventions were more effective when delivering by health professionals than when were delivered by trained teachers (Stallard et al., 2014). Previous studies on the PAUMS SEL program also showed that participants displayed reductions in social anxiety (Coelho et al., 2017; Coelho, Marchante et al., 2015) and social withdrawal (Coelho, Marchante et al., 2015).

However, there are some aspects regarding how universal SEL programs contribute to the reduction of social withdrawal and social anxiety that still require additional studies. For instance, gender differences in participation benefits. One study concluded that boys displayed larger benefits in anxiety from participating in universal interventions than girls (Teubert & Pinquart, 2011). For social anxiety, similar conclusions were reported based on both informants (students and teachers) after participation in a SEL program (Coelho, Marchante et al., 2015). However, a meta-analysis reported mixed results with one study showing greater benefits in anxiety from participating in an SEL program for girls and two others showing no differences regarding gender (Higgins & O’Sullivan, 2015). Finally, in another study, according to self-reports, girls showed greater reductions in social withdrawal and social anxiety after participating in a SEL program, (Coelho et al., 2017).

### School Climate

School climate is defined as the “quality and character of school life” that includes “norms, values, and expectations that support people feeling socially, emotionally, and physically safe.” (Cohen et al., 2009, p. 182). School climate encompasses the social atmosphere of a learning environment in which students have different experiences and includes all aspects of the school experience, including teaching and learning quality, school community relationships, school organization, and the institutional and structural features of the school environment (Wang et al., 2013).

Academic success and student emotional well-being can be facilitated or hindered by the school environment. Evidence suggests that a healthy school climate is associated with several positive outcomes, such as greater student academic achievement, belonging, and engagement (Azpiazu et al., 2023; Bear et al., 2011), better school and socio-emotional adjustment (Bear et al., 2011; Berg & Aber, 2015), and increased life satisfaction (Suldo et al., 2013). Additional studies have indicated that a positive school climate protects students from risk factors such as depression, stress, and anxiety (Lester & Cross 2015), and is associated with fewer conduct problems (Bear et al., 2011) and reduced aggression (Elsaesser et al., 2013).

#### School Climate and SEL Programs

Furthermore, several studies have shown that school climate could positively impact or be impacted by SEL programs. The participation in Positive Action SEL program was associated with decreased school hassle (Stalker et al., 2018), whereas in another study, elementary school students who had more positive perceptions of student–student and teacher–student relationships displayed more positive trajectories in self-esteem after participating in the PAUMS SEL program (Coelho et al. 2020). Moreover, students who participated in the Facing History SEL program described their classrooms as being caring and they felt supported by their teacher (Domitrovich et al., 2022).

#### School Climate and Social Anxiety and Social Withdrawal

A growing body of empirical work has linked school climate to adolescent internalizing concerns (i.e., depression and anxiety), and that anxiety might be more influential in student–student relationships than generalized anxiety (Franco et al., 2022). Furthermore, children who displayed high social anxiety and social withdrawal were more reactive (both positively and negatively) to school climate (Hughes & Coplan, 2018). Also, a more supportive classroom context was associated with smaller increases in social withdrawal (Katulis et al., 2023). However, there are no studies detailing how individual perceptions of school climate dimensions are associated with SEL program’ effectiveness on social withdrawal or social anxiety.

### Positive Attitude Upper Middle School SEL Program and the Gulbenkian Academies for Knowledge Initiative

The PAUMS SEL program has been implemented by the *Académico de Torres Vedras* in the district of Lisbon (first in the municipality of Torres Vedras and later also in the Cadaval municipality). The Project aims to promote children and youth’ healthy behaviors by developing students’ social and emotional competencies. The PAUMS SEL program was developed in alignment with the key competencies identified by CASEL (2022): self-awareness, self-management, social awareness, relationship skills, responsible decision-making and following the recommendations that SEL programs should be sequenced, active, focused, and explicit to maximize results (Durlak et al., 2011). Currently, the program is classroom-based, implemented during the first semester of the school year by a trained psychologist following a manual, and composed of 13 weekly sessions (Marchante & Coelho, 2021). The program is assessed through multi-informants, as recommended by several authors (Achenbach et al., 2008; von der Embse et al., 2023), because different informants see children in different contexts, interact differently with them, and have different mindsets for judging and reporting (Achenbach et al., 2008).

In 2019, this program was selected as a blueprint program for replication in an initiative called the Gulbenkian Academies for Knowledge (GAK). Part of this initiative was aimed to enhance students’ social and emotional competencies by disseminating blueprint Portuguese interventions. Six GAKs replicating the PAUMS SEL program were implemented across Portugal after a two-stage selection process. These Academies spawned seven different municipalities: one in the north (Vizela), one in the center (Pombal), two in Lisbon and the Tagus Valley region (Lisbon and Setúbal), one in the south (including the Faro and Loulé municipalities) and one in the archipelago of Madeira (Caniç̧al).

## Current Study

There is a lack of studies analyzing if SEL programs are effective in reducing social withdrawal and social anxiety and how individual perceptions of school climate may hinder or facilitate SEL program’ effectiveness on social withdrawal and social anxiety. Therefore, the current study has two goals: (1) to analyze the effectiveness of the PAUMS SEL program in reducing social anxiety and withdrawal, when compared to students in the control groups, in a nationwide dissemination implemented under the GAK initiative and (2) to examine whether positive individual perceptions of school climate are associated with positive trajectories of social withdrawal and social anxiety. For the first aim, it was hypothesized that the PAUMS SEL program was effective in reducing social withdrawal (Hypothesis 1) and social anxiety (Hypothesis 2) according to students’ self-reports and teachers’ reports. Based on the literature, it was also hypothesized that, for social withdrawal, girls will benefit more from participating in the PAUMS SEL program than boys (Hypothesis 3), and that for social anxiety the benefits of participating in the PAUMS SEL program will be different by gender (Hypothesis 4). For the second aim, it was hypothesized that individual perceptions of school climate will be associated with program effectiveness. Specifically, it was hypothesized that students’ more positive perceptions of student-student relationships, fairness of rules, school safety, school liking, and teacher-student relationships will be associated with more positive trajectories of social withdrawal (Hypothesis 5) and of social anxiety (Hypothesis 6).

## Methods

### Participants

The sample was sourced from the third wave of a nationwide dissemination of the PAUMS SEL program, under the GAK initiative. This initiative sponsored the replication of programs considered blueprints in SEL programming in Portugal. Originally, 721 middle-school students (from the seventh and eighth grade), who frequented 12 Portuguese public middle schools on the continent and Madeira Archipelago, started the program. However, 17 parents (2.4%) opted out of the classroom assessments, because the program implementation was integrated in a mandatory school subject dedicated to citizenship, but parent could opt out of the assessments.

Therefore, the final sample was composed of 704 upper middle school students from 35 classrooms (ranging from 15 to 27 students, *M*_*classroomsize*_ = 20.11; *SD* = 3.53) who frequented 11 Portuguese secondary public schools. This sample included 367 boys (52.1%) and 335 girls (47.6%); the remaining students classified themselves as “other”. The participants’ mean age was 12.96 (*SD* = 1.09) at the time of the first assessment. Four-hundred-eighty-nine students (69.5%) received the intervention, and 215 students (30.5%) composed the control groups. There was variation in classrooms regarding ethnicity (between 2 and 18% of students were from descended from communities that constitute minorities in Portugal), and socioeconomic status (the percentage of students eligible for free or reduced school meal ranged from 18.2% to 61.8%). Additional information about the participants is displayed in Tables 1,2 and 3.

**Table 1:** Self-reports—descriptive statistics for social anxiety and social withdrawal across times, per gender and modality

	Participants					
	Time 1		Time 2		Time 3	
	*n* = 697		*n* = 684		*n* = 678	
	Social Withdrawal *M* (*SD*)	Social Anxiety *M* (*SD*)	Social Withdrawal *M* (*SD*)	Social Anxiety *M* (*SD*)	Social Withdrawal *M* (*SD*)	Social Anxiety *M* (*SD*)
Boys	5.82 (3.68)	7.24 (3.79)	5.72 (3.70)	6.84 (3.77)	5.49 (3.26)	6.54 (3.30)
Girls	7.14 (4.46)	10.67 (4.29)	7.12 (4.60)	10.39 (4.48)	6.39 (3.89)	9.75 (4.03)
	*t*(693) = −4.26^***^	*t*(693) = −11.19^***^				
Control Group	6.06 (4.01)	8.70 (4.27)	6.39 (4.40)	8.86 (4.26)	6.46 (3.89)	8.99 (4.00)
Intervention Group	6.63 (4.16)	8.97 (4.43)	6.39 (4.13)	8.38 (4.58)	5.70 (3.56)	7.69 (3.95)
	*t*(695) = −1.68	*t*(695) = −0.76				

^***^p < 0.001

**Table 2:** Teacher Reports - Descriptive Statistics for Social Anxiety and Social Withdrawal across times, per Gender and Modality

	Participants			
	Time 1		Time 2	
	*n* = 699		*n* = 694	
	Social Withdrawal *M* (*SD*)	Social Anxiety *M* (*SD*)	Social Withdrawal *M* (*SD*)	Social Anxiety *M* (*SD*)
Boys	3.48 (3.11)	4.45 (2.90)	3.04 (2.97)	4.28 (2.81)
Girls	3.41 (3.09)	4.89 (2.90)	2.90 (3.03)	4.39 (2.74)
	*t*(697) = 0.29	*t*(697) = −2.00^*^		
Control Group	3.66 (2.82)	4.55 (3.03)	3.74 (3.07)	4.83 (3.02)
Intervention Group	3.36 (3.22)	4.70 (2.84)	2.63 (2.90)	4.12 (2.62)
	*t*(697) = 1.18	*t*(697) = −0.81		

^*^p < 0.05

**Table 3:** Descriptive statistics—school climate dimensions across gender and modality

Characteristic	Total (%)	Student-Student Relationships	Fairness of Rules	School Safety	School Liking	Teacher-Student Relationships
		*M* (*SD*)	*M* (*SD*)	*M* (*SD*)	*M* (*SD*)	*M* (*SD*)
Total	697	11.13 (2.10)	8.86 (1.63)	8.92 (1.68)	11.80 (2.30)	21.66 (2.97)
Gender						
Boys	362 (51.9%)	11.35 (2.02)	8.83 (1.67)	8.86 (1.68)	11.66 (2.27)	21.82 (2.86)
Girls	335 (48.1%)	10.90 (2.17)	8.90 (1.58)	8.99 (1.68)	11.97 (2.34)	21.51 (3.08)
		*t*(695) = 2.82^**^	*t*(695) = −0.54	*t*(695) = −0.97	*t*(695) = −1.78	*t*(695) = 1.37
Group						
Control Group	210 (30.1%)	11.47 (1.95)	9.05 (1.58)	9.22 (1.53)	12.32 (2.21)	21.99 (2.87)
Intervention Group	487 (69.9%)	10.99 (2.15)	8.77 (1.64)	8.79 (1.73)	11.58 (2.31)	21.52 (3.00)
		*t*(695) = 2.76^**^	*t*(695) = 2.09^*^	*t*(695) = 3.14^**^	*t*(695) = 3.95^***^	*t*(695) = 1.89

*N* = 697; ^*^p < 0.05; ^**^p < 0.01; ^***^p < 0.001

Regarding additional sources of attrition, the *criteria* followed for excluding students was at the classroom level and not at individual level, i.e., if a student missed one of the three assessments he was kept in the sample, however if an entire classroom missed one of the assessments, all students in that classroom were removed from the sample, this was the case for one 7th-grade classroom (*n* = 23) which had to be removed because there was a change in teachers between T1 and T2, and neither students nor teachers filled out the second assessment. Altogether, 697 students (99%) filled out the first assessment, 684 (97.2%) students filled out the second assessment, and 678 (96.3%) filled out the third assessment. The lower number of students in T3 was due to 26 students having either changed schools or being retained in the same grade at the end of the previous school year and, therefore, they were no longer part of the same classroom. For teacher reports, teachers assessed 699 students (99.3%) in the first moment and 694 (98.6%) in the second moment. Five students were not assessed by their teachers in the first assessment because they were not yet in the classroom at that time; and ten students were not assessed by their teachers in the final assessment because they had already left that classroom (Table 2).

### Measures

#### Social anxiety and social withdrawal—self-reports

The social anxiety and social withdrawal subscales of the Social and Emotional Competences Evaluation Questionnaire (QACSE; Coelho, Sousa, et al., 2015) were used. Both subscales are composed of seven items; social anxiety (e.g., “I get nervous in new group activities”; *α* = 0.78, 0.84 in the present study), and social withdrawal (e.g., “I prefer places that are quiet or isolated”; α = 0.74, 0.81 in the present study). The items are presented as statements, to be rated on a four-point scale (0–*Never*; 1–*Sometimes*; 2–*Frequently;* and 3–*Always*). The questionnaire was validated to be used with adolescents (11 to 16 years), and the instruments’ reliability, validity and factor structure have been validated in two different studies (Coelho, Sousa et al., 2015, Coelho, Sousa et al., 2016) through exploratory and confirmatory factor analysis.

#### Social anxiety and social withdrawal—teacher reports

The social anxiety and social withdrawal subscales of the short form of the Social and Emotional Competences Evaluation Questionnaire - Teacher’s version (QACSE-P-SF; Coelho, Sousa et al., 2016) were used. Both subscales are composed of 5 items; social anxiety (e.g., “He/she look uncomfortable in new group activities”; α = 0.84, 0.83 in the present study); and social withdrawal (e.g., “He/she prefers places that are quiet or isolated”; α = 0.91, 0.88 in the present study). In this instrument, students’ social behavior is evaluated by their head teachers using the same four-point scale as the self-reports. The teacher reports version’ reliability, validity and factor structure were originally validated for use with adolescents in study (Coelho et al., 2014), whereas a later study for the short form employed in this study used confirmatory factor analysis with 657 fourth to ninth graders to expand the instruments’ validity to younger students (Coelho, Sousa et al., 2016).

#### School climate

The Delaware School Climate Survey - Students (DSCS-S) was used. This instrument is composed of 21 items organized into five subscales: student–student relationships (α = 0.77, 0.77 in the present study; e.g., “Students respect other students”); fairness of rules (α = 0.73, 0.78 in the present study; e.g., “School rules are fair”); school safety (α = 0.86, 0.83 in the present study; e.g., “This school is safe”); and school liking (α = 0.76, 0.80 in the present study; e.g., “I like my school”); teacher–student relationships (α = 0.85, 0.81 in the present study; e.g., “I like my teachers”). Each subscale is composed of 3–4 items, except for the teacher-student relationships subscale which is composed of 7 items. Students respond to each item using a 4-point Likert scale (1–Strongly Disagree; 2–Disagree, 3–Agree, and 4–Strongly Agree). Summing scores across items provides a total score (α = 0.92, 0.86 in the current study), with the negatively worded items reverse scored. The factor structure, reliability, and validity of the Portuguese version of the DSCS-S have been well established in previous research (Coelho, Romão et al., 2020).

#### Class Characteristics

Official school records were used as a source of information for class characteristics (i.e., classroom size).

### Procedure

The evaluation took place after obtaining active consent from school boards and parents for the assessment associated with the social and emotional learning program, through consent forms sent in the beginning of the school year. The study was approved by the Ethics Committee of the *Universidade Lusíada* under the project CIPD/2122/DSE/2 and it was conducted following the national professional code of ethics for psychologists. Each Academy in the GAK initiative proposed which schools were to be included to the Gulbenkian Foundation, who approved those proposals. In two (out of four) Academies who participated in this study that included all the upper middle schools in that municipality. Therefore, all 7^th^ and 8^th^ grade classrooms in those schools were enrolled in the program as intervention or waiting list control classrooms. The control group classrooms were randomly selected by each Academy, and they were waitlisted to get the intervention in the next school year. The number of control group classrooms was agreed upon between the Academies and the school boards.

Educational psychologists working for each Academy implemented the questionnaires. These psychologists were the same in all assessments, and these were carried out in regularly scheduled classes and in the presence of the teacher. The students took about 20 minutes per classroom to fill out electronically the questionnaires, using school computers or tablets supplied by the project, thus resulting in no missing data at the individual level. If a student was not present during that period, the psychologist returned the following week (n = 47). Self-report questionnaires for assessing social anxiety and social withdrawal were administered at three different time points: T1 (October 2021) at the beginning of school year and also the beginning of the implementation of the SEL program for a baseline assessment of social anxiety, social withdrawal and school climate; T2 (January/February 2022), in the end of the SEL program for the assessment of social anxiety and social withdrawal after the end of the SEL program; and T3 (September/October 2022), the beginning of the new school year for a follow-up assessment of social anxiety and social withdrawal. Teacher reports were filled out only at T1 and T2, because there are extensive teacher changes between school years.

### Data Analyses

Different analytical strategies were employed for the self- and teacher reports. For teacher reports, because there were only two assessments, repeated measures MANOVAs were conducted to explore potential differential gains from participation in the program, while controlling for gender and grade. This statistical test is recommended for differences between two or more independent groups where participants have repeated measures. The repeated measures 2×2 MANOVA used ‘time’ (pretest vs. posttest) as a within-subjects factor and ‘group (control group vs. intervention group) and ‘gender’ (boys vs. girls) as between-subjects factors, and ‘grade’ (grade 7 vs. grade 8), and ‘classroom size’ as between-subjects’ covariates. Teacher reports were analyzed with the statistical software SPSS22.0 (IBM, 2013).

For self-reports, it was considered that students from the same classroom are much more likely to provide highly correlated responses and that low ICC values for higher level-3 predictors should not deter researchers from using this statistical approach (Bliese et al., 2018). Therefore, because the study used a hierarchical and clustered data set, multilevel linear modeling was employed. Specifically, three-level models were used, because the three assessments were nested within the 704 students, which were nested within 25 school classrooms. Model fit was assessed through the comparison of the function of log-likelihood, using a model deviance test to compare the log-likelihoods. Model fit is better when the difference between models is statistically significant after adjusting for the differences in degrees of freedom, i.e., the second model is significantly smaller than the previous one.

First, to assess the normality assumptions, the distribution of residuals at all three levels was checked by using normal probability plots. The straight-line plots of generated normal scores against the standardized residuals indicated normally distributed residuals. Then, a series of models were created for both outcomes (these are available in the Supplemental materials). In all these models the intercept was used as a random effect. First, an unconditional model (Model 0) that included no predictors was created for the analysis of between-classrooms variance; Model 1 is a growth curve model in which the effect of continuous time on the outcome is treated as linear and allowed to vary across individuals (random slope) to assess within-individual variation. Next, gender and each of the school climate dimensions were entered as explanatory variables at the individual level (Model 2). For Model 3, grade (seventh vs. eighth) and group (control vs. intervention) were entered as explanatory variables at the classroom level. For model 4, a series of cross-level interactions between variables from level 1 (time) and level 2 (individual) were added, namely gender^*^time and schoolclimatedimensions^*^time. In the final models (reported in Table 4), a series of cross-level interactions terms were specified using dummy coding, these cross-level interactions included gender^*^time, and group^*^time. To achieve the goals stated in the second aim, two series (for social withdrawal and social anxiety) of three-way cross-level interactions were added, between group*schoolclimatedimensions*time. All analysis carried out with self-reports to test current study’s hypotheses used MLwiN 3.05.

**Table 4:** Multilevel model analysis final models for self-reports

	Social withdrawal		Social anxiety	
	*β*_0*ijk*_ = 7.08 (0.34)^***^		*β*_0*ijk*_ = 10.27 (0.35)^***^	
	Co-efficient β	SE	Co-efficient β	SE
Classroom				
Grade (if Grade 8)	0.02	0.32	0.41	0.33
Group (if Intervention Group)	0.38	0.35	0.33	0.35
Student				
Gender (if Boys)	−1.33^***^	0.28	−3.42^***^	0.30
Student-Student Relationships	−0.27^**^	0.08	−0.08	0.09
Fairness of Rules	0.18	0.11	0.22	0.12
School Safety	−0.20	0.11	−0.10	0.12
School Liking	−0.20^*^	0.08	0.06	0.09
Teacher-Student Relationships	−0.24^***^	0.07	−0.17^*^	0.07
Time				
Time	0.07	0.13	0.10	0.13
Interactions				
Gender (if Boys) x Time	0.20	0.17	−0.21	0.17
Student-Student Relationships x Time	0.01	0.05	−0.13^*^	0.06
Fairness of Rules x Time	−0.10	0.07	−0.14^*^	0.07
School Safety x Time	0.06	0.07	0.08	0.07
School Liking x Time	−0.05	0.05	−0.06	0.05
Teacher-Student Relationships x Time	−0.09^*^	0.04	0.08	0.05
Grade (if Grade 8) x Time	0.07	0.10	−0.01	0.10
Group (if Intervention Group) x Time	−0.67^***^	0.15	−0.81^***^	0.15
Gender (if Boys) x Group (if IG) x Time	−0.02	0.20	−0.09	0.20
SST x Group (if IG) x Time	0.06	0.06	0.11	0.06
FR x Group (if IG) x Time	0.03	0.08	0.11	0.08
SSF x Group (if IG) x Time	−0.10	0.08	−0.01	0.08
SL x Group (if IG) x Time	0.08	0.06	0.05	0.06
TSR x Group (if IG) x Time	−0.07	0.05	−0.11^*^	0.05
Estimates of Variance Parameters				
Repeated Measures	2.798^***^	0.152	2.537^***^	0.138
Individual Intercept	11.012^***^	0.741	13.136^***^	0.841
Individual Covariance Intercept/Slope	−0.429^*^	0.206	−0.664^**^	0.216
Individual Slope	0.135	0.115	0.343^**^	0.112
Classroom Intercept	0.390^*^	0.184	0.327	0.184
*Deviance* (−2_loglikelihood_)	9699.422		9763.158	
Estimated parameters	29		29	

*CG* Control Group, *IG* Intervention Group, *SST* Student/Student Relationships, *FR* Fairness of Rules, *SSF* School Safety, *SL* School Liking, *TSR* Teacher/Student Relationships

^*^p < 0.05; ^**^p < 0.01; ^***^p < 0.001

## Results

The first aim of the current study was to analyze the effectiveness of the PAUMS SEL program on social withdrawal and social anxiety in a study that analyzed a dissemination of the program. Under that aim, the first two formulated hypotheses assessed program effectiveness, while controlling for individual (gender) and classroom level variables status (grade). For self-reports, the analyses are displayed in Table 4. However, for teacher reports, a preliminary comparative description through a multivariate analysis of group results (control and implementation) through time was implemented; this analysis revealed statistically significant differences, Wilks’ λ = 0.955, *F*(2, 684) = 16.12, *p* < 0.001, η_p_^2^ = 0.045, indicating a beneficial effect for students who participated in the PAUMS SEL program.

### Social Withdrawal

Self-report results for both social anxiety and social withdrawal are displayed in Table 4. After adding all predictors and cross-level interactions, time did not remain a statistically significant predictor of self-reported social withdrawal. Therefore, students did not display statistically significant change in social withdrawal throughout the analyzed period. Individual level statistically significant predictors of self-reported social withdrawal were students’ gender, and the perceptions of student-student relationship, school liking, and teacher-student relationships. As such, boys and students who reported higher levels of student-student relationships, school liking and teacher-student relationships displayed significantly lower levels of social withdrawal. Furthermore, students who reported higher levels of teacher-student relationships also displayed a better trajectory in social withdrawal during the analyzed period, because the cross-level interaction between teacher-student relationships and time was also statistically significant. According to students’ self-reports, there was a statistically significant cross-level interaction between group and time, so hypothesis one was supported by the results, students who participated in the PAUMS SEL program reported a more positive trajectory in social withdrawal than control groups. However, hypothesis three was not supported by the results, because the three-way cross-level interaction—between gender, group, and time—was not statistically significant, therefore girls did not benefit more from participating in the PAUMS SEL program. Finally, hypothesis five was not supported by the results because none of the three-way cross-level interactions—between group, individual perceptions of school climate, and time—were statistically significant. Therefore, more positive perceptions of school climate dimensions were not associated with a better effectiveness of the PAUMS SEL program on social withdrawal, during the analyzed period.

Regarding the effectiveness of the PAUMS SEL program on social withdrawal, the results from teacher reports are mostly aligned with the self-reports. Results showed a significant interaction effect (time x group), with intervention groups displaying a more positive trajectory in social withdrawal than control groups, *F*(1,685) = 17.54, *p* < 0.001, η_p_^2^ = 0.025, thus also supporting hypothesis one. Furthermore, no statistically significant main effects were found in any dimension, for neither time nor group. However, teachers reported statistically significant differential program effects by gender, as the results showed a significant interaction effect between time, group and gender in social withdrawal, *F*(1, 685) = 5.36, *p* = 0.021, η_p_^2^ = 0.008, girls benefited more than boys, thus supporting hypothesis three.

### Social Anxiety

For students reporting on their own social anxiety, after adding all predictors and cross-level interactions, the within-individual predictor (time) was not a statistically significant predictor of social anxiety. Therefore, according to the students, social anxiety remained stable throughout the analyzed period. As displayed in Table 4, the other individual and cross-level statistically significant predictors of self-reported social anxiety were gender, teacher-student relationships and two cross-level interactions—between student-student relationships and time, and between fairness of rules and time. Boys had lower social anxiety than girls, and students who reported higher levels of teacher-student relationships also had lower social anxiety. Higher levels of student-student relationships and fairness of rules were associated with a significant decrease in their social anxiety during the analyzed period. Results also showed that intervention group students displayed a larger decrease in their social anxiety when compared with control group students, confirming hypothesis two. Furthermore, hypothesis four was not supported by the results, because there was not a statistically significant three-way cross-level interaction between gender, group, and time. Therefore, students did not report differential effects by gender from participating in the program in their trajectories of social anxiety. Finally, hypothesis six was partially supported by the results. Only one of the three-way cross-level interactions—between group, individual perceptions of school climate and time—was associated with a better effectiveness of the PAUMS SEL program on social anxiety. Specifically, intervention group students with higher levels of teacher-student relationships displayed a statistically significant steeper decrease in their social anxiety during this period.

The results from teacher reports were consistent with self-reports regarding the effectiveness of the PAUMS SEL program on social anxiety. After adjusting for gender and grade, results showed a significant interaction effect (time x group) with intervention groups displaying a more positive trajectory in social anxiety, *F*(1,685) = 26.70, *p* < 0.001, η_p_^2^ = 0.008, supporting hypothesis two. There were no statistically significant main effects, neither for time nor group. Furthermore, the teachers did not report differential program effects by gender; the results did not show a statistically significant interaction effect between time, group and gender in social anxiety, *F*(1, 685) = 0.34, *p* = 0.562, η_p_^2^ = 0.000; thus rejecting hypothesis four.

## Discussion

There is strong evidence that universal SEL programs have positive impacts on social and emotional competences, behavioral change, and educational outcomes (Durlak et al., 2022; Taylor et al., 2017), and several authors have emphasized positive impact of universal SEL programs on mental health (Clarke et al., 2021; Durlak et al., 2022; Stallard et al., 2014). However there is a lack of studies focusing specifically on SEL programs’ effectiveness on social withdrawal and social anxiety, and under different perceived school climates. Therefore, the current study analyzed whether the PAUMS SEL program reduced social withdrawal and social anxiety in a nationwide study, and if more positive perceptions of school climate were associated with more positive trajectories of social withdrawal and social anxiety for the period analyzed.

For social withdrawal, the results show that both informants reported that participating in the PAUMS SEL program led to a better trajectory for intervention groups students compared to control group students, thus supporting the first hypothesis. There are considerably less studies analyzing the effectiveness of SEL programs on social withdrawal, compared to social anxiety, but the results from the current study are aligned with Coelho, Marchante et al. (2015), where both informants had reported the students in the intervention group displayed a more positive trajectory in social withdrawal, and they contradict Coelho et al. (2017) where no statistically significant results were found in self-reports. The results also support Clarke et al. (2021) who had argued for the benefits on mental health from social and emotional competencies enhancement.

For social anxiety, the results were also consistent between students and teachers: both reported statistically significant positive trajectories in social anxiety for students that participated in the PAUMS SEL program compared with the control group students. Therefore, the second hypothesis was supported by the results, which were similar to previous results conducted with smaller and less heterogeneous samples (Coelho et al., 2017; Coelho, Marchante et al., 2015). They are also aligned with reports by several authors (Stallard et al., 2014; Teubert & Pinquart, 2011) who had found positive effects of SEL programs on anxiety.

Although both students and teachers reported that girls displayed initial higher social withdrawal and social anxiety, in self-reports there were no statistically significant differences between genders in terms of benefiting from the PAUMS SEL program. However, teacher reports identified greater benefits for girls from program participation. Therefore, the results partially negate the third and fully negate the fourth hypotheses. The results mostly highlight the universal nature of the PAUMS SEL program, and they are consistent with the existing literature (Durlak et al., 2022), although they are not aligned with previous studies. In previous studies, boys and girls displayed different gains in anxiety or social anxiety from participating in universal programs (Teubert & Pinquart, 2011; Coelho et al., 2017; Coelho, Marchante et al., 2015), sometimes favoring boys (Coelho, Marchante et al., 2015; Teubert & Pinquart, 2011) and other times favoring girls (Coelho et al., 2017). These results can be attributed to the smaller sample sizes previous studies used, which may be susceptible to specific characteristics and dynamics (Durlak et al., 2022), whereas the current study used a heterogeneous nationwide sample and thus provides a broader view of universality of the PAUMS SEL program.

Regarding the influence of perceived school climate on the effectiveness of this SEL program, none of the perceived school climate dimensions were associated with improved trajectories in social withdrawal, thus rejecting hypothesis five. However, intervention group students who reported more positive perceptions of teacher-student relationships displayed a steeper decrease in social anxiety than intervention group students with lower perceptions of teacher-student relationships, therefore partially supporting hypothesis six. The results of the current study are aligned with previous literature (Hughes & Coplan, 2018; Katulis et al., 2023), who had reported the relevance of school climate for students with high social anxiety. Furthermore, one dimension of perceived school climate (teacher-student relationships) was associated with reduced school withdrawal, and several dimensions of perceived school climate (student-student relationships and fairness of rules) were associated with reduced social withdrawal. These results provide additional support to existing studies that suggest that more positive perceptions of school climate perceptions are associated with less social anxiety (Franco et al., 2022). Two other conclusions can be drawn from this outcome. First, given that in a previous study with younger students (Coelho, Bear et al., 2020) more positive perceptions of student–student and teacher–student relationships were associated with more positive trajectories in self-esteem, positive school climate may be more important for younger students regarding SEL programs’ effectiveness. Second, during SEL program participation, more positive perceptions of school climate may be positively associated with improvements in student social and emotional competencies but not associated with reductions in social withdrawal and social anxiety.

Overall, the results consistently suggest that students and teachers reported that the PAUMS SEL program had a positive effect on social withdrawal and social anxiety. The relevance of the current results is heightened due to the use of a broader sample that encompassed a wide range of schools with accounting for different individual perceptions of school climate. Even with this wide range of school-climate perceptions, only one school climate dimension (teacher-student relationships) was associated with differences in the effectiveness of the PAUMS SEL program on social anxiety for the period analyzed, which reinforced the importance of establishing positive teacher-student relationships, as proposed by Domitrovich et al. (2022), as a complementary approach to help to maximize the impact of a SEL program on social anxiety of students. Therefore, the current study provides support for the effectiveness of the PAUMS SEL program on social withdrawal and social anxiety across genders, and on social withdrawal in differently perceived school climates.

Contrarily to students’ self-reports—which had three assessments available, it was only possible to collect teacher reports for two time points, which limited the available choice of statistics because it is not advisable to apply growth curve analysis with just two time points. Therefore, when confronted with different results between self-reports and teacher reports—as was the case with social withdrawal—the former were prioritized, because they are more reliable as they describe the trajectory of the social anxiety and social withdrawal over a longer period. However, future studies should match assessment moments for students and their teachers.

Furthermore, teachers were not blind to the status of the students in the intervention vs. control groups, which may have influenced the results. Also, because of the methodological limitations detailed above, it was not possible to use teacher reports to analyze the influence of school climate on the effectiveness of the PAUMS SEL program. However, given that individual perceptions of school climate were being used, it was more relevant to analyze self-reports to analyze the influence of school climate perceptions.

Future studies should analyze the relationships between the key competencies proposed by the CASEL (2022) and the social withdrawal and social anxiety trajectories—i.e., if the reduction in social withdrawal and social anxiety is associated with significant increases in self-control and/or social awareness.

## Conclusion

There is a lack of studies focusing specifically on SEL programs’ effectiveness on social withdrawal and social anxiety, and how their effectiveness may vary under differently perceived school climates. The results of the current study showed that the PAUMS SEL program was effective in reducing social withdrawal and social anxiety in a nationwide sample, and they showed the relevance of positive teacher-student relationships as they are associated with higher program effectiveness on social anxiety, highlighting the importance for adolescents to establish healthy and positive relationships with adults. These results add to the current literature by clarifying that universal school based SEL programs contribute to the promotion of mental health and positive development of adolescents by reducing social withdrawal and social anxiety, which is particularly relevant in the post COVID-19 pandemic context where there was an increase in mental health difficulties, including social anxiety and social withdrawal.

## Supplementary Information

Supplementary Information
