# Supplementary material for: Can an Universal School-Based Social Emotional Learning Program Reduce Adolescents’ Social Withdrawal and Social Anxiety?
Source: J Youth Adolesc. 2023 Aug 17;52(11):2404–16. doi: 10.1007/s10964-023-01840-4 (PMC10495480; doi:10.1007/s10964-023-01840-4)
Supplement: Supplementary file 1 — Supplementary Information [file 10964_2023_1840_MOESM1_ESM.docx]

Supplementary Table 1 - *Multilevel Model Analysis Models for Social Withdrawal*

| Parameters | Model 0  Null | Model 1  Level 1: Time |  | Model 2  Level 2: Individual | Model 3  Level 3: Class | Model 4  Cross-Level Interactions  Level 1 and 2 |
| --- | --- | --- | --- | --- | --- | --- |
| Estimates of Fixed Effects | | | | | | |
| Intercept | 6.26 (0.18)^***^ | 6.54 (0.19)^***^ |  | 7.10 (0.21)^***^ | 7.47 (0.32)^***^ | 7.56 (0.33)^***^ |
| Time |  | -0.28 (0.05)^***^ |  | -0.28 (0.05)^***^ | -0.28 (0.05)^***^ | -0.37 (0.07)^***^ |
| Gender (if boys) |  |  |  | -1.13 (0.26)^***^ | -1.12 (0.26)^***^ | -1.30 (0.29)^***^ |
| Student-Student Relationships |  |  |  | -0.22 (0.08)^**^ | -0.22 (0.08)^**^ | -0.29 (0.08)^***^ |
| Fairness of Rules |  |  |  | 0.10 (0.10) | 0.10 (0.10) | 0.18 (0.11) |
| School Safety |  |  |  | -0.20 (0.10) | -0.21 (0.10)^*^ | -0.20 (0.11) |
| School Liking |  |  |  | -0.17 (0.08)^*^ | -0.18 (0.08)^*^ | -0.21 (0.08)^*^ |
| Teacher-Student Relationships |  |  |  | -0.20 (0.06)^**^ | -0.20 (0.06)^**^ | -0.24 (0.07)^***^ |
| Grade (if Grade 8) |  |  |  |  | -0.11 (0.31) | -0.10 (0.31) |
| Group (if Intervention Group) |  |  |  |  | -0.46 (0.33) | -0.46 (0.33) |
| Gender (if boys) x Time |  |  |  |  |  | 0.16 (0.10) |
| SSR x Time |  |  |  |  |  | 0.06 (0.03) |
| FR x Time |  |  |  |  |  | -0.07 (0.04) |
| SS x Time |  |  |  |  |  | -0.01 (0.04) |
| SL x Time |  |  |  |  |  | 0.03 (0.03) |
| TSR x Time |  |  |  |  |  | 0.03 (0.02) |
|  | | | | | | |
| Estimates of Covariance Parameters | | |  |  |  |  |
| Repeated Measures | 3.178 (0.122)^***^ | 2.840 (0.154)^***^ |  | 2.801 (0.152)^***^ | 2.801 (0.152)^***^ | 2.800 (0.152)^***^ |
| Individual Intercept | 12.300 (0.735)^***^ | 13.738 (0.888)^***^ |  | 11.071 (0.744)^***^ | 11.175 (0.749)^***^ | 11.120 (0.746)^***^ |
| Individual Covariance Intercept/Slope |  | -0.780 (0.231)^***^ |  | -0.513 (0.211)^*^ | -0.558 (0.212)^**^ | -0.504 (0.209)^**^ |
| Individual Slope |  | 0.256 (0.120)^*^ |  | 0.268 (0.119)^*^ | 0.268 (0.119)^*^ | 0.223 (0.117) |
| ClassRoom Intercept | 0.872 (0.267)^**^ | 0.824 (0.260)^**^ |  | 0.502 (0.198)^*^ | 0.406 (0.186)^*^ | 0.400 (0.185)^*^ |
| ICC | .053 | .047 |  | .037 | .029 | .028 |
| *R^2^* (between-individuals) |  |  |  | .100 | .091 | .096 |
| *R^2^* (between-classes) |  |  |  | .392 | .514 | .541 |
|  | | | | | | |
| *Deviance* (-2_loglikelihood_) | 10000.716 | 9953.456 |  | 9761.781 | 9759.670 | 9741.365 |
| Δ-2LL |  | 47.260 ^***^ |  | 191.675^***^ | 2.111 | 18.305^**^ |
| Number of estimated parameters | 5 | 7 |  | 13 | 15 | 21 |

*Note.* ^*^*p* < .05; ^**^*p* < .01; ^***^*p* < .001; SST = Student/Student Relationships; FR = Fairness of Rules; SSF = School Safety; SL = School Liking; TSR = Teacher/Student Relationships

Supplementary Table 2 *- Multilevel Model Analysis Models for Social Anxiety*

| Parameters | Model 0  Null | Model 1  Level 1: Time |  | Model 2  Level 2: Individual | Model 3  Level 3: Class | Model 4  Cross-Level Interactions  Level 1 and 2 |
| --- | --- | --- | --- | --- | --- | --- |
| Estimates of Fixed Effects | | | | | | |
| Intercept | 8.50 (0.17)^***^ | 8.90 (0.18)^***^ |  | 10.66 (0.22)^***^ | 10.83 (0.33)^***^ | 10.91 (0.33)^***^ |
| Time |  | -0.41 (0.05)^***^ |  | -0.41 (0.05)^***^ | -0.41 (0.05)^***^ | -0.48 (0.07)^***^ |
| Gender (if boys) |  |  |  | -3.28 (0.28)^***^ | -3.24 (0.28)^***^ | -3.39 (0.30)^***^ |
| Student-Student Relationships |  |  |  | -0.12 (0.08) | -0.13 (0.08) | -0.09 (0.09) |
| Fairness of Rules |  |  |  | 0.15 (0.11) | 0.15 (0.11) | 0.15 (0.11) |
| School Safety |  |  |  | -0.01 (0.11) | -0.01 (0.11) | -0.11 (0.12) |
| School Liking |  |  |  | 0.05 (0.08) | 0.04 (0.08) | 0.04 (0.09) |
| Teacher-Student Relationships |  |  |  | -0.16 (0.06)^*^ | -0.16 (0.06)^*^ | -0.17 (0.07)^*^ |
| Grade (if Grade 7) |  |  |  |  | 0.38 (0.31) | 0.37 (0.31) |
| Group (if Intervention Group) |  |  |  |  | -0.59 (0.33) | -0.59 (0.33) |
| Gender (if boys) x Time |  |  |  |  |  | -0.13 (0.10) |
| SSR x Time |  |  |  |  |  | -0.03 (0.03) |
| FR x Time |  |  |  |  |  | -0.06 (0.04) |
| SSF x Time |  |  |  |  |  | 0.09 (0.04)^*^ |
| SL x Time |  |  |  |  |  | 0.01 (0.03) |
| TSR x Time |  |  |  |  |  | 0.01 (0.02) |
|  | | | | | | |
| Estimates of Covariance Parameters | | |  |  |  |  |
| Repeated Measures | 3.204 (0.123)^***^ | 2.527 (0.137)^***^ |  | 2.539 (0.138)^***^ | 2.539 (0.138)^***^ | 2.538 (0.138)^***^ |
| Individual Intercept | 15.052 (0.886)^***^ | 16.446 (1.018)^***^ |  | 13.172 (0.844)^***^ | 13.332 (0.852)^***^ | 13.305 (0.850)^***^ |
| Individual Covariance Intercept/Slope |  | -0.857 (0.245)^***^ |  | -0.757 (0.226)^***^ | -0.826 (0.228)^***^ | -0.806 (0.226)^***^ |
| Individual Slope |  | 0.510 (0.119)^***^ |  | 0.508 (0.119)^***^ | 0.508 (0.119)^***^ | 0.488 (0.118)^***^ |
| ClassRoom Intercept | 0.612 (0.244)^*^ | 0.684 (0.248)^**^ |  | 0.603 (0.207)^**^ | 0.333 (0.185) | 0.339 (0.186) |
| ICC | .032 | .035 |  | .037 | .021 | .021 |
| *R^2^* (between-individuals) |  |  |  | .125 | .114 | .116 |
| *R^2^* (between-classes) |  |  |  | .015 | .456 | .446 |
|  | | | | | | |
| *Deviance* (-2_loglikelihood_) | 10133.537 | 10037.775 |  | 9835.901 | 9832.215 | 9824.776 |
| Δ-2LL |  | 95.762^***^ |  | 201.874^***^ | 3.686 | 7.349 |
| Number of estimated parameters | 5 | 7 |  | 13 | 15 | 21 |

*Note.* ^*^*p* < .05; ^**^*p* < .01; ^***^*p* < .001; SST = Student/Student Relationships; FR = Fairness of Rules; SSF = School Safety; SL = School Liking; TSR = Teacher/Student Relationships
